# Ultrasonography Elastography to Predict the Diagnosis, Severity, and Treatment Indication of Esophageal Varices in Patients with Chronic Liver Diseases

**DOI:** 10.3390/diagnostics15151867

**Published:** 2025-07-25

**Authors:** Azusa Wada, Yasunobu Yamashita, Mikitaka Iguchi, Yoshiyuki Ida, Takao Maekita, Reiko Ashida, Masayuki Kitano

**Affiliations:** Second Department of Internal Medicine, Wakayama Medical University, Wakayama 641-0012, Japan; wasao4218@gmail.com (A.W.); mikitaka@wakayama-med.ac.jp (M.I.); y-mori@wakayama-med.ac.jp (Y.I.); maekita@wakayama-med.ac.jp (T.M.); rashida@wakayama-med.ac.jp (R.A.); kitano@wakayama-med.ac.jp (M.K.)

**Keywords:** elastography, esophageal varices, shear wave, F-index

## Abstract

**Background/Objectives**: Esophageal varices (EVs) are a serious complication of liver cirrhosis. Guidelines for cirrhosis/chronic liver diseases (CLDs) do not specify a follow-up period or the need for esophagogastroduodenoscopy (EGD). EGD is a useful but uncomfortable procedure for the assessment of varices. Follow-up with abdominal ultrasonography (AUS) is recommended in patients with CLDs. If EVs are assessed by AUS, more patients eligible for endoscopic screening of EVs can be selected. We aimed to investigate whether AUS elastography [shear wave (Vs) and F-index] can predict the diagnosis, severity, and treatment indication of EVs. **Methods**: Between April 2018 and October 2022, we retrospectively collected data of 194 patients who underwent elastography and EGD for CLDs. The correlations between Vs/F-index values and presence/severity of EVs were evaluated. Each cut-off value for diagnosis and treatment indication of EVs was investigated. **Results**: 85 patients without exclusion criteria were enrolled. Vs and F-index values were significantly higher in patients with EVs than in patients without EVs (*p* = 0.0005 and 0.0021, respectively) and positively correlated with severity of EVs. The cut-off Vs and F-index values for the presence of EVs were 1.63 m/s and 1.88, respectively, with 88.1%/83.3% sensitivity, 48.8%/51.2% specificity, and 0.71/0.70 area under the curve (AUC). The cut-off Vs and F-index values for treatment indication were 1.71 m/s and 2.08, respectively, with 100%/88.2% sensitivity, 45.6%/52.9% specificity, and 0.69/0.70 AUC. There were no significant differences between the two modalities. **Conclusions**: Elastography may provide objective assessment and thus be a non-invasive screening tool for diagnosis and treatment indication of EVs.

## 1. Introduction

Liver cirrhosis is the final stage of chronic liver disease. In this stage, the liver is changed into an irreversible replacement of liver parenchyma with fibrotic tissue and regenerative nodules. Liver cirrhosis due to chronic liver diseases is a health concern worldwide. Liver cirrhosis has various etiologies, including hepatitis B virus (HBV) infection, hepatitis C virus (HCV) infection, alcohol intake, nonalcoholic fatty liver disease, and autoimmune liver diseases [1]. Liver cirrhosis has clinical findings such as jaundice, ascites, hepatic encephalopathy, bleeding (e.g., variceal hemorrhage) due to portal hypertension, or infections. Although esophageal varices (EVs) are a serious complication of liver cirrhosis, endoscopic treatment can be prevented by bleeding them [2]. The estimated prevalence of EVs in patients with cirrhosis has been reported to be 50% [3], and the mortality rate of variceal bleeding ranges from 20% to 35% [4]. According to the Baveno V consensus guidelines, screening endoscopy is recommended for all patients with cirrhosis to identify those who should undergo prophylactic treatment [5]. Although endoscopic screening is the most effective method for detecting varices, it is invasive and difficult to perform in some patients and in all medical institutions. Therefore, non-invasive accurate methods for predicting the diagnosis and severity of varices are needed.

Among imaging modalities, ultrasonography is the most non-invasive method and is performed in all settings including clinics. Moreover, it is a necessary tool for the screening of hepatocellular carcinoma in patients with chronic liver diseases (CLDs). Ultrasound-based liver elastography is a new technique for measuring tissue stiffness. According to the European Association for the Study of the Liver guidelines, a liver biopsy is not necessary for the determination of the liver fibrosis stage including cirrhosis, and non-invasive methods such as ultrasound-based elastography and magnetic resonance (MR) elastography liver elastography are recommended [6]. MR elastography is also useful for evaluating liver fibrosis, but ultrasound-based elastography is preferential to MR elastography especially in centers lacking access to standard MRI-based elastography [7]. Therefore, ultrasonography-based elastography is used in clinical practice as a non-invasive indicator of fibrosis assessment, replacing liver biopsy. EVs are complications of long-standing portal hypertension due to factors such as fibrous tissue in patients with liver cirrhosis. Therefore, using elastography to diagnose fibrosis may be useful for diagnosis of EVs. The presence of portal vein embolization is also a risk of EVs. Therefore, ultrasound is also useful for assessment of the information about the blood flow in the portal vessels using Doppler imaging. However, the present report is limited in its evaluation of the liver stiffness measurement with ultrasound. There are two types of ultrasound-based liver elastography techniques: strain elastography and shear wave elastography (Vs). The F-index, which is a new concept, is determined using a combinatorial method of Vs and strain elastography. There are no reports of diagnosis of varices with the F-index. The aim of this study was to evaluate whether Vs as a conventional method and the F-index can predict the diagnosis and treatment indication of EVs.

## 2. Patients and Methods

### 2.1. Study Design

This study was approved by the ethics committee of Wakayama Medical University (No. 3823). It was performed in accordance with the ethical standards laid down in the 2013 Declaration of Helsinki and its later amendments. The primary outcome was evaluation of the abilities of two elastography techniques (Vs/F-index) to predict the diagnosis and treatment indication of EVs. Secondary outcomes were measurement of the correlations between Vs/F-index values and presence/severity of EVs.

### 2.2. Study Population

Between April 2018 and October 2022, this study retrospectively enrolled 194 patients who underwent two elastography techniques (Vs/F-index), esophagogastroduodenoscopy (EGD), and computed tomography (CT) for CLDs. Exclusion criteria were as follows: (1) age < 17 years, (2) duration between ultrasound-based liver elastography and EGD/CT >3 months, (3) previous history of treatment for EVs such as endoscopic variceal ligation and endoscopic injection sclerotherapy, (4) previous history of surgical treatment of the stomach or esophagus, (5) presence of portal vein embolization or a shunt, (6) percentage of the net amount of effective shear wave velocity (VsN, %) ≤ 50, and (7) presence of gastro-renal shunts on CT.

### 2.3. EGD

All patients underwent EGD (GIF-260 and GIF-290; Olympus, Tokyo, Japan) to assess the presence and severity of EVs by endoscopists who had performed at least 1000 procedures. EGD was performed in the left lateral position with the monitoring of heart rate, saturation, and blood pressure.

### 2.4. Definition of EVs

Endoscopic findings of EVs were assessed according to the Japan Society of Portal Hypertension guidelines from 2022 [8], which consist of six items: location, form, color, red color (RC) sign, bleeding sign, and mucosal finding. Forms of varices (F) were divided into F0 (after treatment and no recurrence), F1 (straight and small-caliber varices), F2 (tortuous veins with a bead-like appearance), and F3 (tumor-shaped varices). RC sign was classified as RC0, RC1 (few, localized), RC2 (between RC1 and RC3), and RC3 (many in all the circumference). The presence of EVs was defined as F1, F2, and F3, regardless of RC sign. The treatment indication for EVs was defined as varices with F1 in addition to a positive RC sign, F2, or F3 [9,10,11].

### 2.5. Ultrasonography Procedure

For all patients, an ultrasonography observation system (ARIETTA 850; FULIFILM Healthcare, Tokyo, Japan) was used. Prior to performing elastography, the morphology of the liver, gallbladder, and spleen, and the presence of ascites were evaluated in the supine position using conventional B-mode imaging in each patient.

### 2.6. Vs

The region of interest (ROI) used for elastographic evaluation was manually selected. The ROI (10 × 20 mm) was set at liver segment 6 or 7 through the right intercostal space without vessels, intrahepatic bile ducts, cysts, or abnormal lesions at a depth of 1.5–2.0 cm below the capsule of the liver. Shear wave velocity is a quantitative method used to determine absolute tissue elasticity. Vs was measured five times, and the median value was determined. The calculated VsN is used to assess whether the Vs value is reliable. A VsN > 50% indicates that the data are highly reliable [12]. Therefore, data with VsN ≤ 50% were excluded to ensure reliability.

#### F-Index

The F-index was calculated as follows: F-index = −12.259 − 0.0199 × mean relative strain value − 0.0069 × standard deviation of relative strain value + 0.0068 × percentage of low strain area − 0.0214 × complexity of low strain area − 0.0772 × skewness + 0.0862 × kurtosis + 1.6659 × entropy − 1.3422 × inverse difference moment + 42.1004 × angular second moment + 0.0011 × contrast + 8.2076 × correlation + 1.3869 × Vs [13].

### 2.7. Statistical Analysis

The Chi-square test for qualitative variables and the Mann–Whitney U test for quantitative variables were used to compare categorical variables. Dunnett’s test was used to compare Vs/F-index values between patients with and without EVs, different form grades of EVs, and with and without treatment indication for EVs according to the Japan Society of Portal Hypertension guideline 2022 [8]. Correlations between Vs/F-index values and the severity of EVs were evaluated using Spearman’s correlation. The diagnostic accuracies of Vs and the F-index for EVs were calculated from the receiver operating characteristics (ROC) curves, with the maximum Youden index used to determine cut-off points, and sensitivity and specificity were calculated. The area under ROC (AUROC) was defined as low (0.5 to <0.7), moderate (0.7 to <0.9), or high (≥0.9) accuracy. All statistical analyses were performed using JMP Pro version 13 (SAS Institute Inc., Cary, NC, USA). A *p*-value < 0.05 was considered statistically significant.

## 3. Results

**Figure 1:**
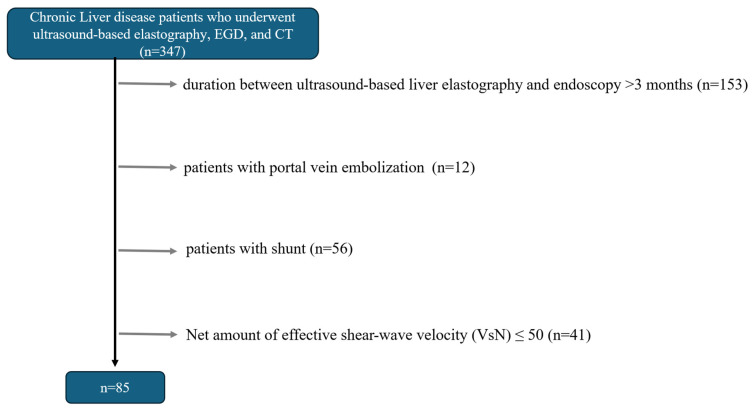
Flowchart of selection of the study population.

According to the exclusion criteria, 85 patients were enrolled in this study (Figure 1). The mean patient age was 68.9 ± 12.4 years, and the mean platelet count was 16.9 ± 7.4 (×10^4^/µL). Of these patients, 47 were men. Final diagnoses were chronic hepatitis B (four patients), chronic hepatitis C (28 patients), alcoholic hepatitis (16 patients), nonalcoholic steatohepatitis (15 patients), autoimmune hepatitis (six patients), and primary biliary cholangitis (10 patients). A total of 36 hepatocellular carcinomas (size, 19 (8–120) mm; location (S2, 3; S3, 5; S4, 4; S5, 6; S6, 8; S7, 5; S8, 5) were identified in 31 cases. Of the 85 patients, 42 had EVs. The clinical, biochemical, and endoscopic characteristics of patients with and without EVs are summarized in Table 1.

**Figure 2:**
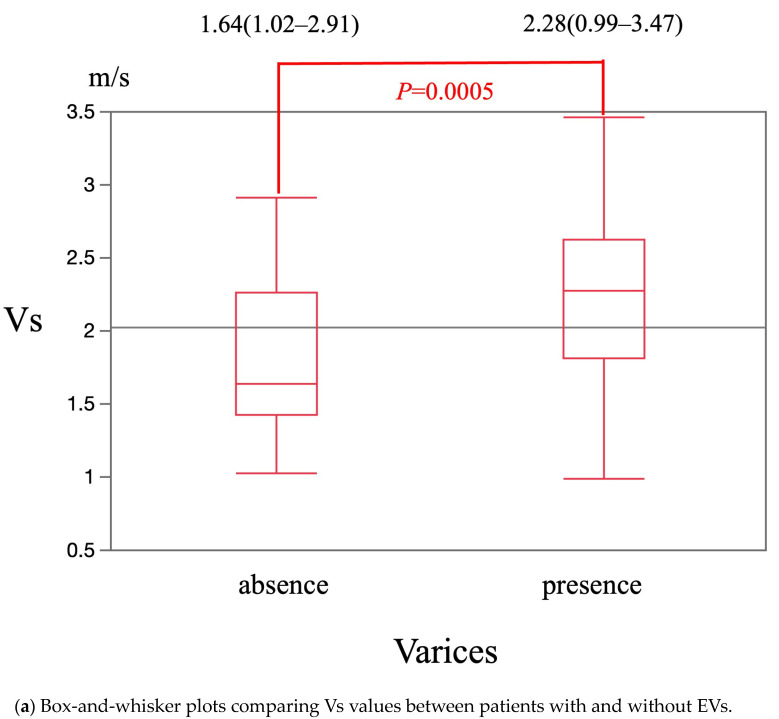

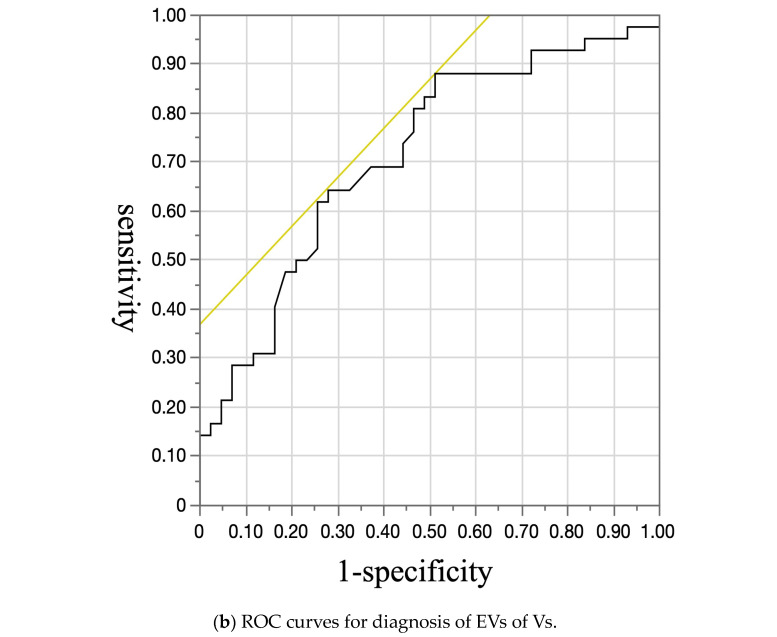
The diagnostic performance of Vs in presence or absence of EVs.

**Figure 3:**
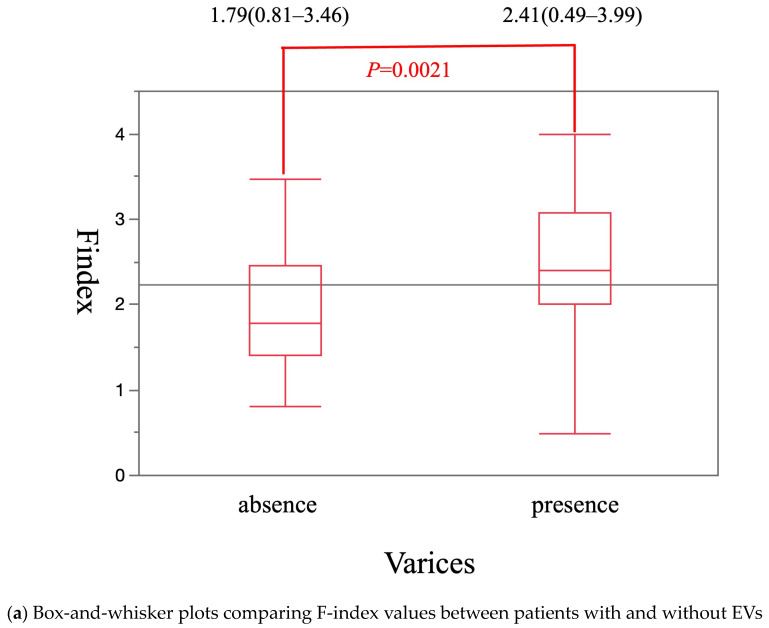

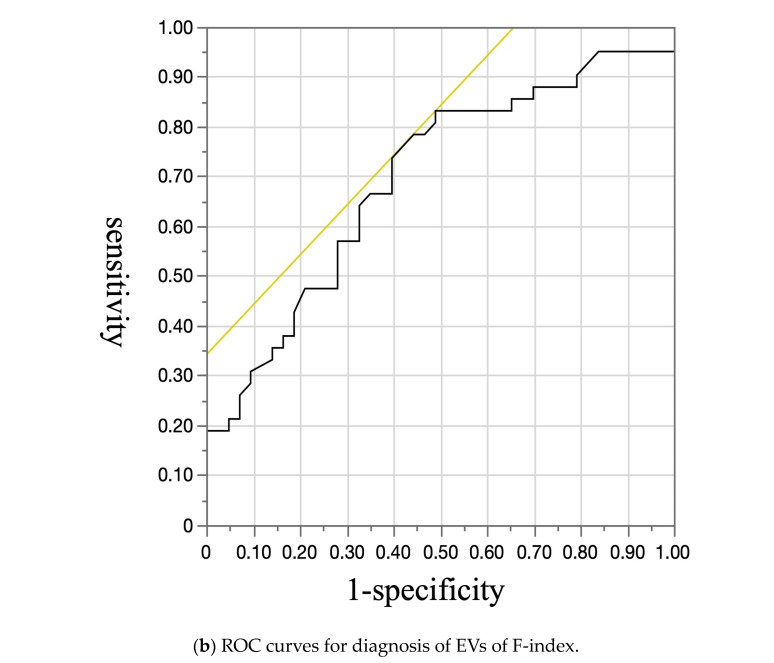
The diagnostic performance of F-index in presence or absence of EVs.

The median Vs value was significantly higher in patients with EVs [2.28 m/s (0.99–3.47 m/s)] than in patients without EVs [1.64 m/s (1.02–2.91 m/s)] (*p* = 0.0005) (Figure 2a). The cut-off Vs value for diagnosis of EVs was 1.63 m/s. The sensitivity, specificity, and area under the receiver operating characteristics curve (AUROC) were 88.1%, 48.8%, and 0.71, respectively (Figure 2b). The median F-index value was significantly higher in patients with EVs [2.41 (0.49–3.99)] than in patients without EVs [1.79 (0.81–3.46)] (*p* = 0.0021) (Figure 3a). The cut-off F-index value for the diagnosis of EVs was 1.88. The sensitivity, specificity, and AUROC were 83.3%, 51.2%, and 0.70, respectively (Figure 3b).

**Figure 4:**
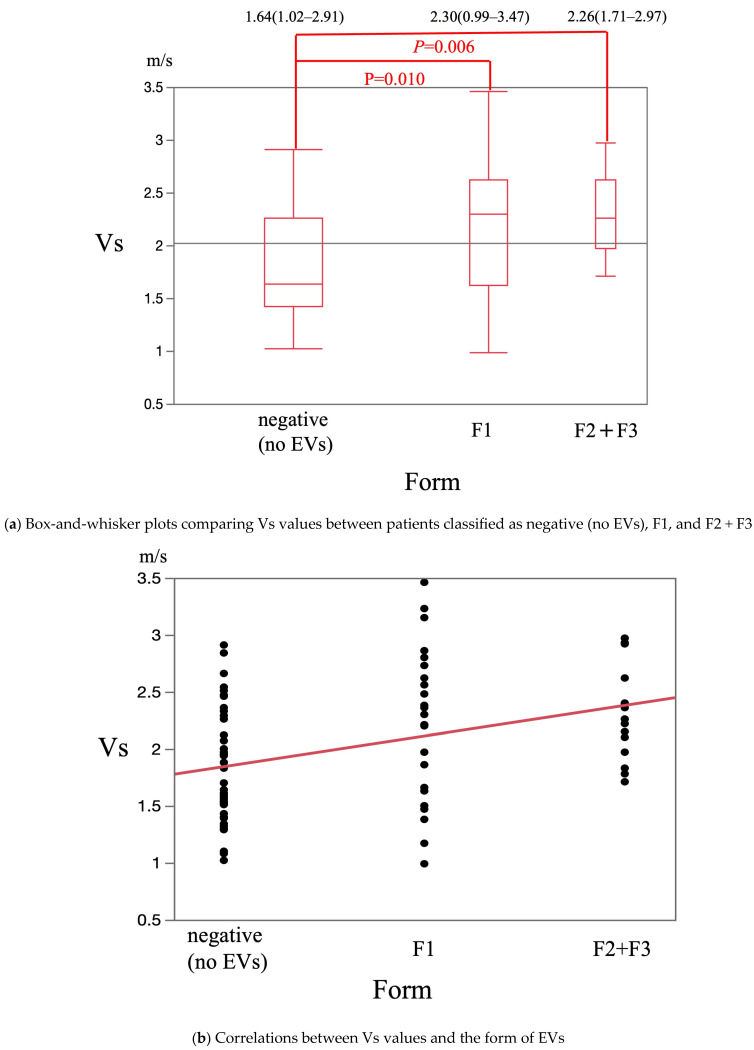
The correlation between EVs morphology and Vs.

**Figure 5:**
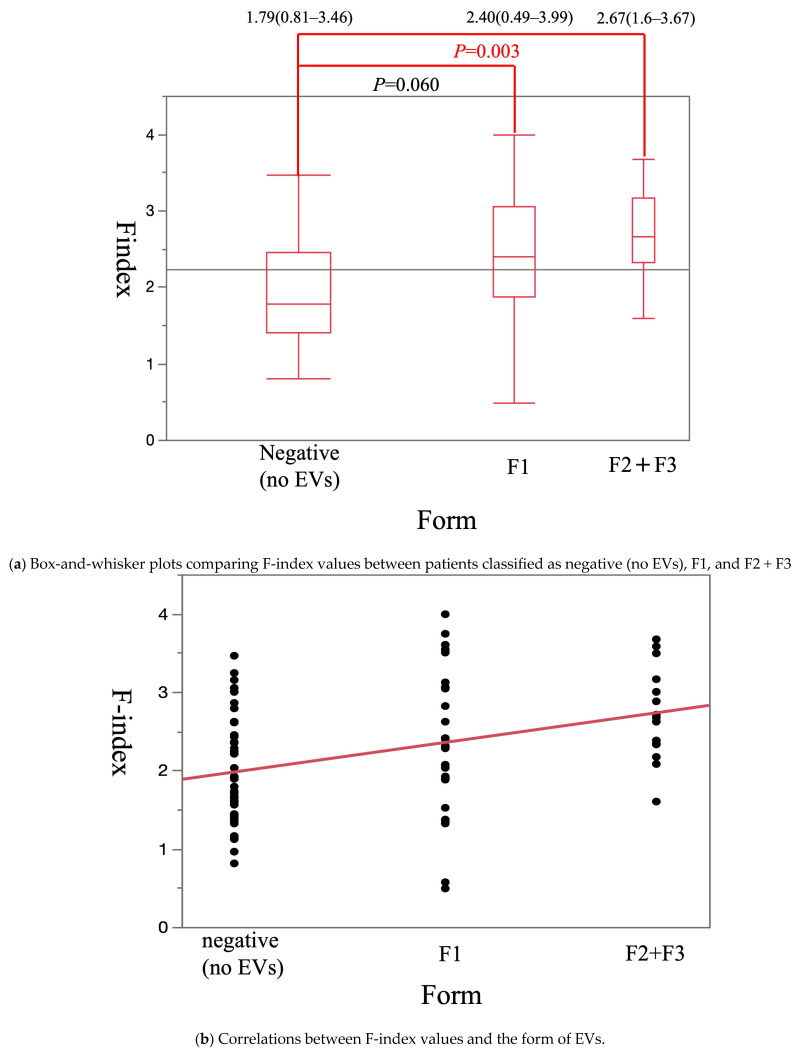
The correlation between EVs morphology and F-index.

The median Vs values in patients classified as negative (no EVs), F1, and F2 + F3 were 1.64 m/s (1.02–2.91 m/s), 2.30 m/s (0.99–3.47 m/s), and 2.26 m/s (1.71–2.97 m/s), respectively. This was significantly higher in patients classified as F1 and F2 + F3 than in patients classified as negative (*p* = 0.01 and *p* = 0.006, respectively) (Figure 4a). The Vs value was significantly and positively correlated with the F stage (*p* < 0.001) (Figure 4b). The median F-index values in patients classified as negative, F1, and F2 + F3 were 1.79 (0.81–3.46), 2.40 (0.49–3.99), and 2.67 (1.60–3.67), respectively. It was significantly higher in patients classified as F2 + F3 than in patients classified as negative (*p* = 0.003) (Figure 5a) and tended to be higher in patients classified as F1 than in patients classified as negative (*p* = 0.06) (Figure 5a). The F-index value was significantly and positively correlated with the F stage (*p* < 0.001) (Figure 5b).

**Figure 6:**
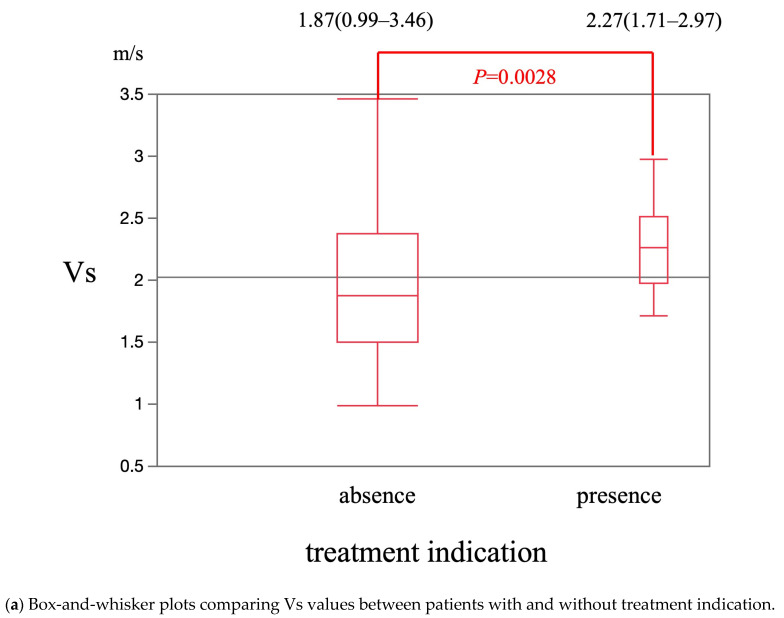

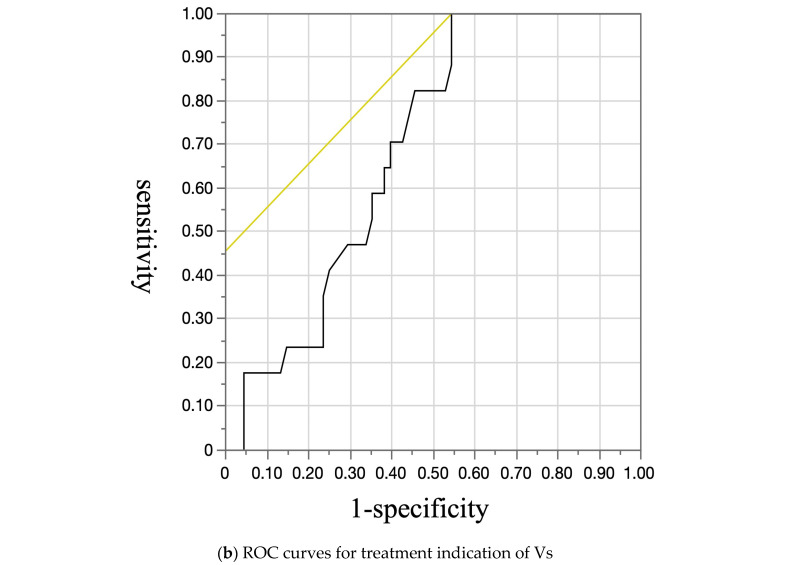
The diagnostic performance of Vs for treatment indication.

**Figure 7:**
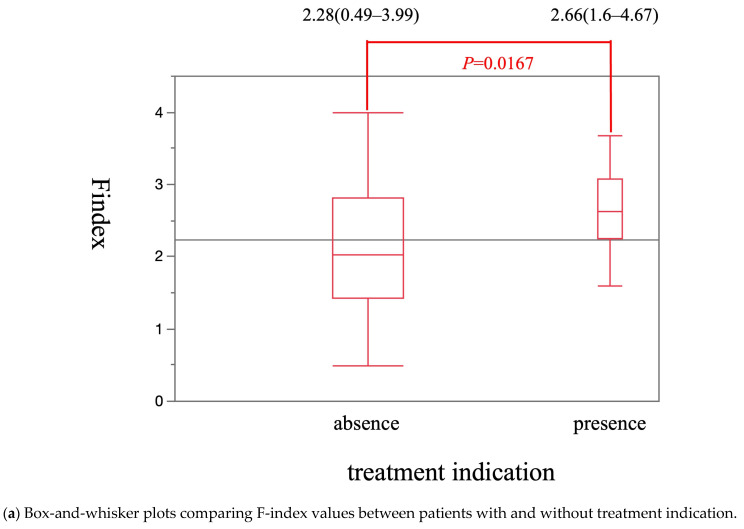

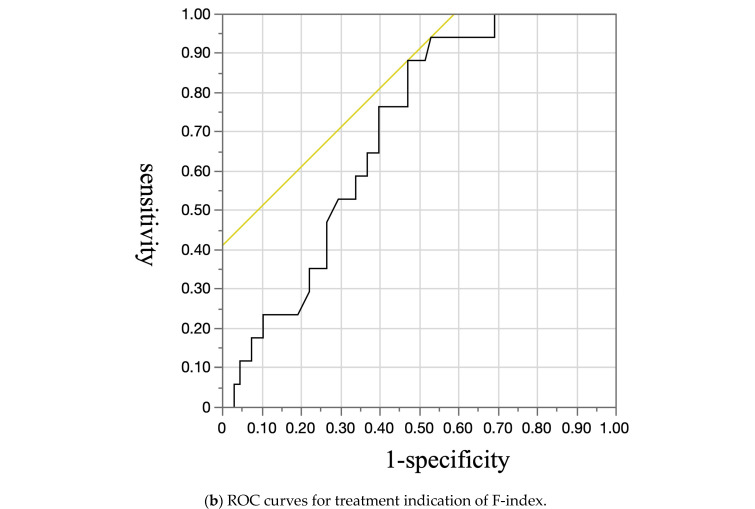
The diagnostic performance of F-index for treatment indication.

The median Vs value was significantly higher in patients with treatment indication [2.27 m/s (1.71–2.97 m/s)] than in patients without treatment indication [1.87 m/s (0.99–3.46 m/s)] (*p* = 0.0028) (Figure 6a). The cut-off Vs value for treatment indication was 1.71 m/s. The sensitivity, specificity, and AUROC were 100%, 45.6%, and 0.69, respectively (Figure 6b). The median F-index value was significantly higher in patients with treatment indication [2.66 (1.60–4.67)] than in patients without treatment indication [2.28 (0.49–3.99)] (*p* = 0.0167) (Figure 7a). The cut-off F-index value for treatment indication was 2.08. The sensitivity, specificity, and AUROC were 88.2%, 52.9%, and 0.70, respectively (Figure 7b).

In the diagnosis and treatment indication of EVs, there was no difference between Vs and the F-index.

## 4. Discussion

The prevalence of EVs in cirrhotic patients is variable; therefore, universal endoscopic screening would result in a large number of unnecessary endoscopies and place a heavy burden on endoscopy units. The present study revealed that endoscopy was unnecessary for approximately half of patients without EVs. Therefore, compliance with screening programs may be hampered by the perceived unpleasantness of endoscopy. The endoscopy has some sever complications such as perforation, bleeding, and cardiopulmonary adverse [14]. Predicting the presence of EVs by a non-invasive method might increase compliance and would help to restrict the performance of endoscopy to patients with a high probability of having EVs. Moreover, in terms of prophylactic treatment, it is important to detect varices with treatment indication in order to prevent hemorrhage.

On the other hand, guidelines recommend ultrasonography, for screening the degree of fibrosis in patients with CLDs and hepatocellular carcinoma because fibrosis is an important risk factor for hepatocarcinogenesis [6,15,16,17,18]. In another report, the measurement of liver stiffness has prospects for assessing fibrotic changes in the liver according to typical ranges of biomechanical properties. Therefore, EUS elastography may be useful for the prediction of hepatic cell activities and correlation with hepatocellular carcinoma [19]. Therefore, ultrasonography with elastography is performed for screening of hepatocellular carcinoma and the degree of liver fibrosis in patients with CLDs. If abdominal ultrasonography performed for screening of hepatocellular carcinoma can be used to detect EVs, it will be a non-invasive screening method for EVs. Therefore, there are some reports regarding prediction of EVs with elastography [20,21,22,23,24,25,26]. However, these studies investigated shear wave imaging as a non-invasive diagnostic method. In previous reports [27,28,29], shear wave imaging is simple to perform but can be difficult when inflammation, jaundice, and congestion are present. Therefore, it is difficult to diagnose liver fibrosis using shear wave imaging alone in cases with mild-to-moderate fibrosis [13].

Strain imaging is a modality for color mapping of relative tissue distortion and can diagnose liver fibrosis in patients with inflammation, jaundice, or congestion [21,28,29,30] but is highly dependent on measurement techniques and requires operator training. Therefore, combinatorial use of strain and shear wave imaging (F-index) is more useful for diagnosis of hepatic fibrosis. Yada et al. reported that the diagnostic ability of a combination of shear wave and strain elastography (F-index) for fibrosis is higher than that of a conventional method (shear wave) [31,32]. On the other hand, in terms of prevention, it is also important to detect varices with treatment indication.

Therefore, we employed the F-index to assess the severity of fibrosis in patients with CLDs. To the best of our knowledge, no previous study has evaluated the F-index for prediction of EVs. The present study demonstrated correlations between the absolute Vs/F-index values and morphology of EVs (severity). The abilities of both Vs and the F-index to predict the diagnosis and treatment indication of EVs were moderate. On the other hand, the AUROC did not differ between Vs and the F-index, suggesting that the F-index was as same as Vs in abilities to predict the diagnosis and treatment indication of EVs. In the present study, there may have been few patients with mild-to-moderate fibrosis, which may have contributed to the lack of a significant difference between the two modalities. There may have been many patients with advanced liver disease and severe fibrosis because this study was conducted at a high-volume liver disease center. A larger multicenter study is needed to investigate which modality is better to detect EVs.

This study has several limitations. First, it was a retrospective single-center study enrolling a small number of patients. Second, many of the patients had advanced liver disease at the high-volume liver disease center. Further studies incorporating a larger number of patients from multiple centers are required. Third, they produced a fluctuation data of elastography might occur in chronic hepatitis B because it was not as stable as other chronic liver diseases.

## 5. Conclusions

Elastography (shear wave and F-index) provides objective assessment and can thus be a non-invasive screening tool for predicting the diagnosis, severity, and treatment indication of EVs.

## Figures and Tables

**Table 1:** Characteristics of the patients.

Variable	Esophageal Varices		*p*-Value
	Absent (*n* = 43)	Present (*n* = 42)	
Sex (male/female)	19/24	28/14	0.0371
Age (years)	70.47 ± 11.20	67.26 ± 13.41	
Etiology			
HBV	0	4	0.0382
HCV	16	12	0.3969
Alcoholic hepatitis	4	12	0.0231
NASH	8	7	0.8147
AIH	5	1	0.0961
PBC	8	2	0.0477
Others	2	4	0.3805
Child-Pugh			
A	39	34	0.1970
B	4	8	0.1970
C	0	0	
Presence of hepatocellular carcinoma	17	14	0.5526
Presence of ascites	2	7	0.0719
Platelet count (×10^4^/µL)	19.85 ± 6.2358	13.83 ± 7.315	<0.0001

HBV: hepatitis B virus, HCV: hepatitis C virus, NASH: nonalcoholic steatohepatitis, AIH: autoimmune hepatitis, PBC: primary biliary cholangitis. Significant *p* values are highlighted in red.

## Data Availability

Data are available from the corresponding author upon reasonable request, subject to institutional and privacy restrictions.
